# Sex- and age-differences in blood manganese levels in the U.S. general population: national health and nutrition examination survey 2011–2012

**DOI:** 10.1186/1476-069X-13-87

**Published:** 2014-10-24

**Authors:** Youssef Oulhote, Donna Mergler, Maryse F Bouchard

**Affiliations:** Department of Environmental and Occupational Health, Université de Montréal, Montréal, Canada; CHU Sainte-Justine Research Center, Montréal, Canada; CINBIOSE, Université du Québec à Montréal, Montréal, Canada

**Keywords:** Blood manganese, Sex, Age, Population survey, NHANES

## Abstract

**Background:**

Manganese is an essential element, but excessive manganese exposure has neurotoxic effects.

**Objective:**

To examine blood manganese levels in the general population with respect to sex, age, race/ethnicity, pregnancy and menauposal status, as well as levels of trace elements in blood.

**Methods:**

We used data from the National Health and Nutrition Examination Survey, a national survey of U.S. residents (n = 7720 participants, ages 1 to 80 years). General linear models and generalized additive models were used to examine the association between blood manganese concentration and participants’ characterisics, accounting for the complex survey design.

**Results:**

Blood manganese levels ranged from 1.6 to 62.5 μg/L, with arithmetic means of 10.6 and 9.2 μg/L for women and men, respectively. The following characteristics were significantly associated with higher blood manganese levels: female sex, younger age, Asian origin, and being pregnant. In addition, there were non-linear relationships between blood manganese levels and cadmium, iron, lead, and mercury levels.

**Conclusion:**

The higher blood manganese levels observed among females suggest sex-related metabolic differences in the regulation of manganese, and elevated levels among pregnant women suggest an important role of manganese in reproduction. The present study supports the need to take into consideration age- and sex-related differences in blood manganese levels, as well as pregnancy status when examining manganese essentiality or toxicity.

## Background

Manganese (Mn) is widespread in the environment [1]. It is an essential element required for the functioning of many enzymes and involved in oxidative stress protection, as well as in the formation of connective tissue and bone. Upon overexposure, however, Mn can cause severe neurologic impairment, ranging from mild neurobehavioral deficits to manganism, a debilitating neurodegenerative disease resembling Parkinson’s disease [2]. Blood Mn levels are often used as a bioindicator of exposure to this metal in epidemiological studies investigating Mn toxicity [3–6], but little data are available on levels of blood Mn in the general population.

Mn is an essential nutrient with complex homeostatic mechanisms regulating its absorption, disposition, and biliary excretion. These mechanisms tend to maintain the optimal circulating manganese levels, which might vary depending on the sex and age of individuals, among other factors. Indeed, studies indicate that blood Mn levels vary differently in males and females and over the lifespan. For instance, levels are higher during infancy, than during adulthood, and women have slightly higher levels than men [7]. Blood Mn concentration increases during pregnancy, and levels at the end of gestation are three to four times higher than in non-pregnant women [8]. In addition, individuals with low iron status have higher Mn blood levels [9–12], possibly because of the upregulation of mechanisms of gastrointestinal absorption shared by iron and Mn [13].

The interest for Mn toxicity developed from adverse health effects observed in occupationally-exposed individuals. Occupational exposure occurs in certain industrial processes such as welding, ferroalloy and smelting operations, and Mn mining [14]. For the general population, the main source of Mn is usually the diet, but environmental exposures can occur in populations living near industrial Mn emissions [15, 16], from Mn-containing pesticides [17, 18], and from the consumption of water with naturally-occurring elevated Mn levels [19, 20]. Finally, some medical conditions are associated with high Mn body burden, such as liver diseases that impair biliary excretion [21], and long-term parenteral nutrition with excessive Mn supplementation [22].

For the first time since the U.S. National Health and Nutrition Examination Survey (NHANES) program started in the seventies, blood Mn levels were measured in 2011–2012. The main objective of the present study is to examine predictors of blood Mn levels in the general U.S. population.

## Methods

### Study design and population

The NHANES is a cross-sectional, population-based health survey of non-institutionalized U.S. residents conducted by the National Center for Health Statistics (NCHS) of the Centers for Disease Control and Prevention (CDC). The NHANES uses a complex, multistage probability sampling design, with oversampling of certain subgroups. Participants completed individual and household surveys, which included questions about demographics and health history, and blood and urine samples were collected during a physical examinations at mobile centers. The study protocol is described in detail elsewhere [23]. The NHANES was approved by the NCHS institutional review board, and all participants provided written informed consent.

### Mn and other elements in blood

Whole blood specimens were frozen (−30°C), stored, and shipped for analysis to the Division of Laboratory Sciences, National Center for Environmental Health of the CDC. Whole blood Mn levels were measured in participants 1 year and older, using inductively coupled plasma mass spectrometry (ICP-MS). All values were above the limit of detection. Other metals were also measured in blood by ICP-MS: lead, cadmium, mercury and selenium. Finally, iron (non-heme) concentration was measured in blood using the DcX800 method, but this measure was only performed in individuals ages older than 12 years. Further methodological details on the laboratory analyses are described elsewhere [24]. The limits of detection (LOD) were 0.25 μg/dL for lead, 30 μg/L for selenium, 1.06 μg/L for Mn, and 0.16 μg/L for cadmium and mercury. Mn and selenium were detected in all participants, 1%, 7% and 30% of individuals had measurements below LOD respectively for lead, mercury, and cadmium levels, and were replaced by LOD/√2.

### Participant characteristics

Data was collected on several variables by questionnaire and during the examination, including: age, race/ethnicity, education (available for those 20 years and older), poverty/income ratio (PIR (the ratio of self-reported family income to the family’s appropriate threshold value), grouped into quartiles), length of time in the U.S. for those not born in the country, and BMI. For children 2–19 years, BMI was grouped into underweight (<5^th^ percentile), normal weight (5^th^ to <85^th^ percentiles), overweight (85^th^ to <95^th^ percentiles), and obese (≥95^th^ percentile). For adults, BMI was grouped into underweight (<18.5), normal weight (18.5 to 25), overweight (>25 to 30), and obese (>30). Pregnancy status was available only for females between 20 and 44 years of age, and was based on pregnancy test and self-report. Postmenauposal women were identified as those not having menstruated in the last 12 months, because of either menopause or hysterectomy. Some data were missing for education (n = 2), PIR (n = 502), and BMI (n = 219).

In addition, questionnaire data was used to identify participants with a history of liver problems. Of the 7920 participants, we excluded 200 individuals with a history of liver problems. Since this information was available only for individuals ages ≥20 years, we assumed that individuals below 20 years were free of liver disease.

### Statistical analysis

In multivariable analyses, we used general linear models to identify the predictors of blood Mn concentrations in the whole group of participants. We also ran age-stratified analyses (<12 years, n = 1761/≥12 years, n = 5959) because different factors may predict blood Mn levels in younger and older individuals (and because serum iron concentration was available only for individuals ≥12 years). Models for all participants and for younger individuals (<12 years) included the following variables: age (years), sex, race/ethnicity, PIR, and blood levels of lead, cadmium, mercury and selenium. In addition to these variables, the models for individuals ages 12–80 years included BMI and blood iron concentrations. We also ran sex-stratified analyses for both age groups. We included education in a model restricted to individuals ages 20 years and older (n = 4720). Finally, the models to assess menopausal and pregnancy status as predictors of Mn levels were restricted to women ages 20 years and older (n = 2411), and women ages 20–44 years, respectively. These two models included age, race/ethnicity, PIR, and blood levels of lead, cadmium, mercury and selenium as covariates.

Blood levels of lead, cadmium, mercury, selenium, and iron approximated a lognormal distribution, and were log-transformed (base 10) to address skewness and outliers’ effect. In a sensitivity analysis, we re-ran models using imputed data for missing PIR and BMI. The imputation was done using multiple imputations by chained equations (MICE), an approach that uses all the variables in the models to impute the missing values [25]. Furthermore, we ran generalized additive models (GAM) with penalized smoothing regression splines to examine the shape of relationship between Mn levels and other trace elements in blood.

We used the survey [26] package in R [27] to obtain estimates of association and confidence intervals accounting for the multistage probability sampling design of the NHANES. We also used the weights to adjust for the oversampling of certain population subgroups and account for nonresponse and non-coverage in the NHANES. All tests were 2-sided and p <0.05 was the level of significance.

## Results

Table 1 shows the characteristics of the 7720 individuals ages 1 to 80 years forming the study group, and mean blood Mn levels by characteristics. Most of the participants were non-Hispanic white (63.6%) and overweight or obese (60.2%); 51% were female. Blood Mn levels ranged from 1.6 to 62.5 μg/L, with an arithmetic mean of 9.9 μg/L (95% CI: 9.8-10.1 μg/L) and a geometric mean (GM) of 9.3 μg/L. The distribution of blood Mn concentrations was 5.5, 7.5, 9.2, 11.4, and 16.6 μg/L for the 5^th^, 25^th^, 50^th^, 75^th^, and 95^th^ percentiles, respectively (Table 2).

**Figure 1:**
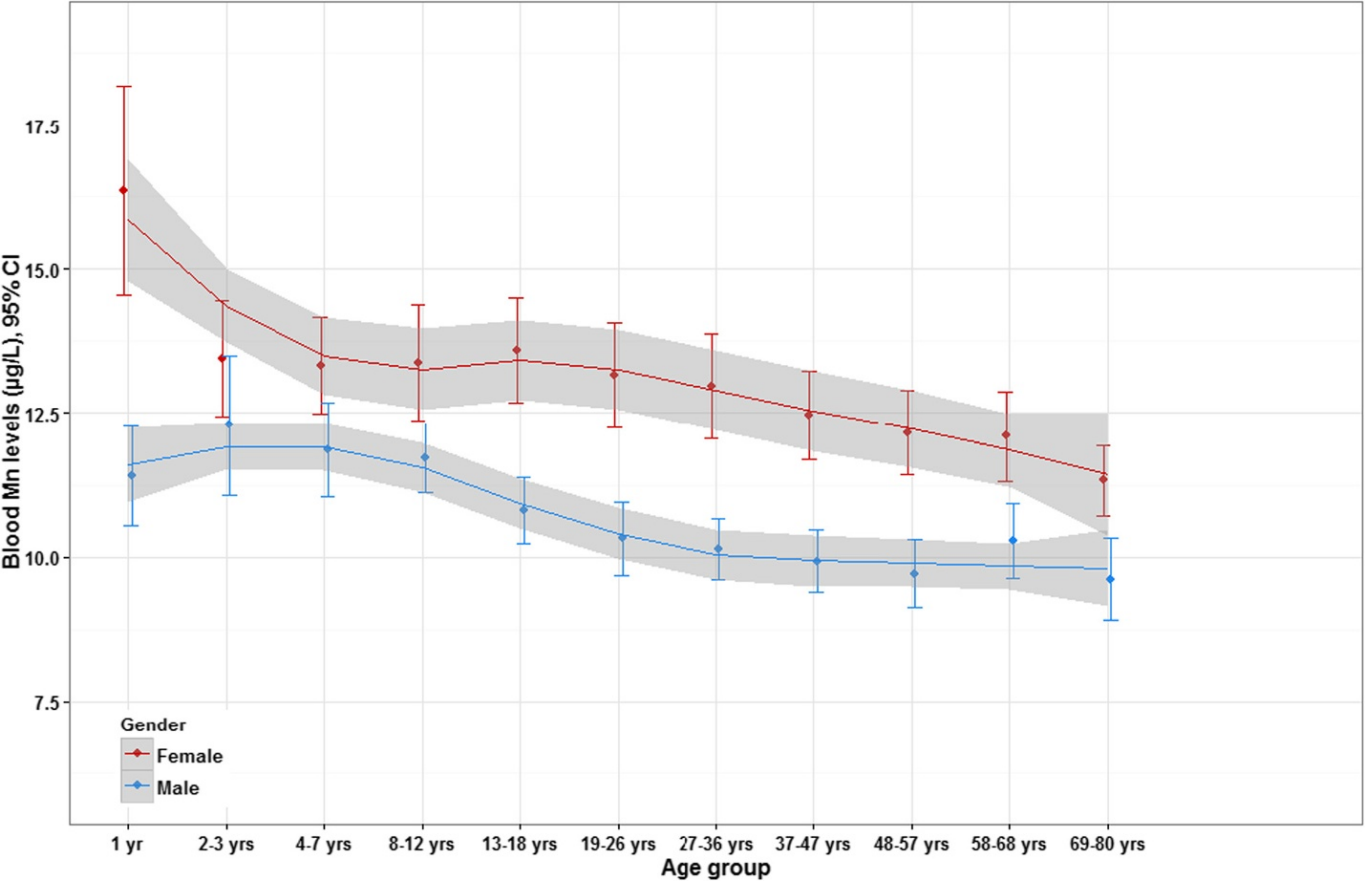
**Estimated mean blood Mn levels (95% CI) with respect to sex and age, adjusted for race/ethnicity and blood cadmium levels (n = 7720).** Statistics are weighted.

**Figure 2:**
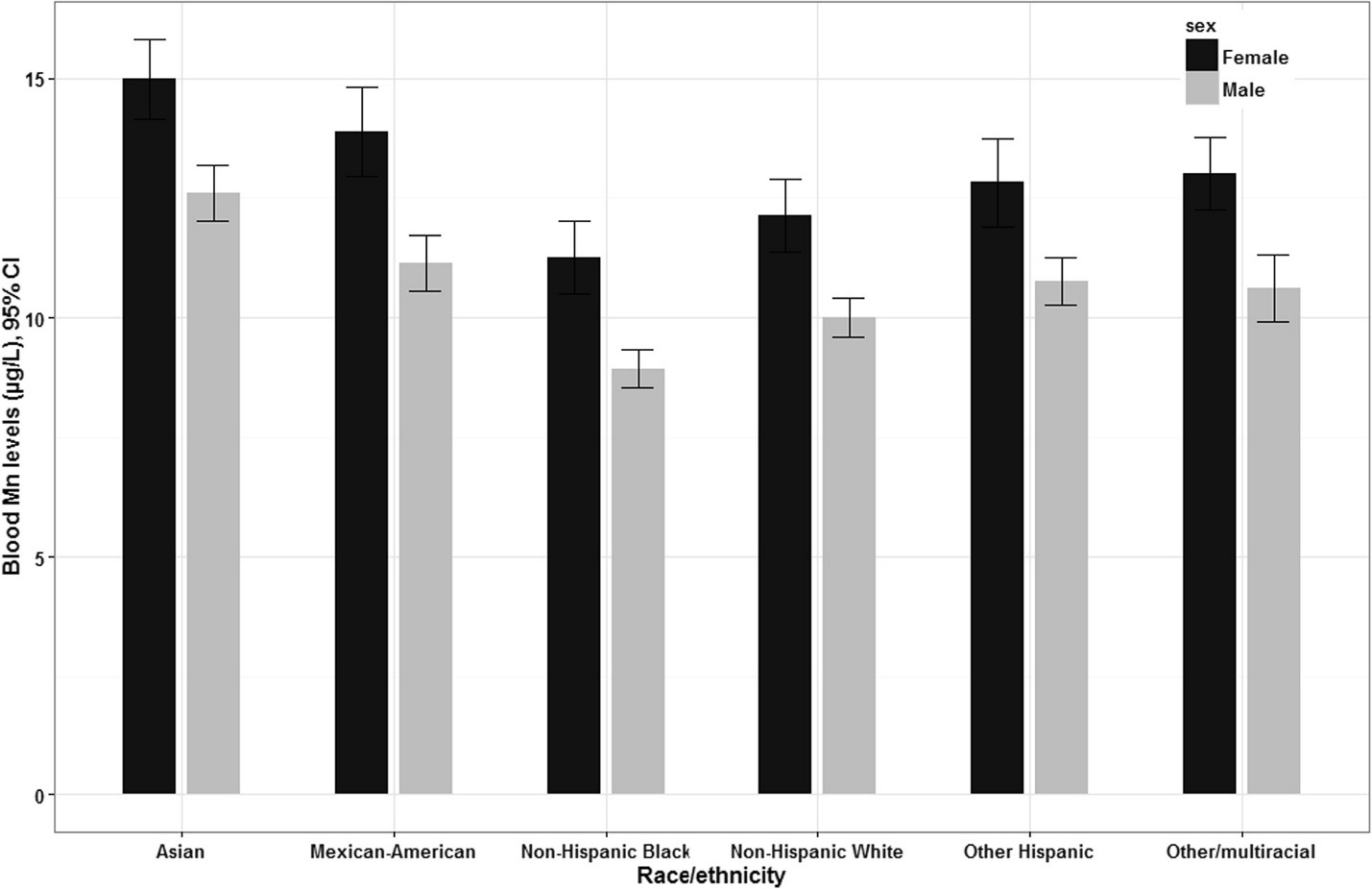
**Estimated mean blood Mn levels (95% CI) with respect to sex and race/ethnicity, adjusted for age and blood cadmium levels (n = 7720).** Statistics are weighted.

In models including participants from the entire age range, the following characteristics were significantly associated with higher blood Mn levels: female sex, younger age, Asian origin, and higher cadmium blood levels. There was no relation between blood Mn levels and PIR, mercury, selenium, and lead in blood. Figure 1 shows blood Mn level with respect to age and sex, and adjusted for race/ethnicity and blood cadmium levels. Females had levels higher than males at all ages. Among females, levels were the highest at 1 year old, decreased sharply at 2–3 years and remained stable during childhood (4 to 12 years), increased slightly during teenage years (13–18 years) and decreased steadily afterwards. Among males, levels remained fairly stable between 1 and 12 years, decreased afterward and then remained stable after 36 years of age. Figure 2 shows blood Mn levels with respect to sex and race/ethnicity, and adjusted for age and blood cadmium levels. Using the non-Hispanic whites as the reference group, all other race/ethnicity groups had significantly different mean Mn levels (p < 0.05). The highest levels were found in individuals of Asian origin, and the lowest among non-Hispanic Black individuals. Blood Mn levels were higher in females than in males among all race/ethnicity groups.

**Figure 3:**
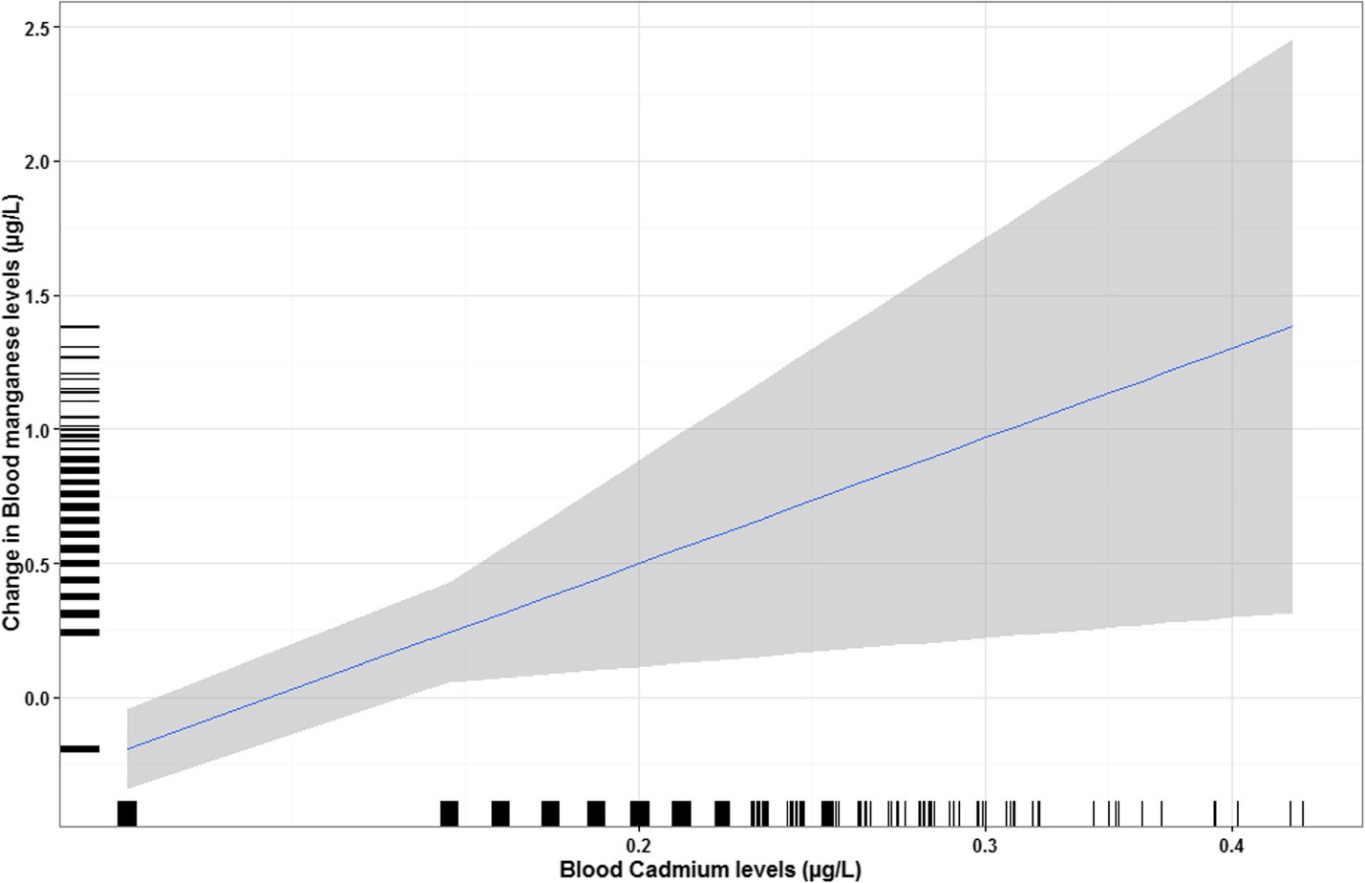
**Association between blood Mn and cadmium levels (95% CI) for individuals 1–11 years, adjusted for age, sex, race/ethnicity, and blood lead, selenium, and mercury levels (n = 1761).** Statistics are weighted.

In age-stratified analyses, blood Mn levels were significantly associated with race/ethnicity and blood cadmium levels in individuals below 12 years; for race/ethnicity, the same pattern of results was observed as shown in Figure 2 (highest in individuals of Asian origin and lowest among non-Hispanic Black individuals). In addition, girls had higher levels than boys but not significantly (p = 0.09). The relationship between blood Mn and cadmium levels was linear as revealed by GAM analyses (Figure 3). PIR, blood mercury, lead and selenium were not associated with blood Mn levels (all at p > 0.3). In sex-stratified analyses, age and cadmium were marginally associated with blood Mn levels in girls (p < 0.1), whereas race/ethnicity remained significantly associated with blood Mn in both girls and boys.

**Table 1:** **Study group characteristics and blood Mn levels (μg/L) in participants ages 1 to 80 years; weighted statistics, NHANES 2011-2012 (n=7720)**

Characteristics	n	Weighted %	GM blood Mn (μg/L)	AM blood Mn (μg/L)	95% CI
Total	7720	100	9.3	9.9	(9.8, 10.1)
Sex					
Male	3857	48.7	8.7	9.2	(9.0, 9.4)
Female	3863	51.3	9.9	10.6	(10.4, 10.8)
Age					
<12 years	1761	12.0	10.4	10.9	(10.6, 11.2)
12-80 years	5959	88.0	9.2	9.8	(9.6, 9.9)
Education^a^					
Less than 9th grade	453	5.6	9.2	10.0	(9.4, 10.5)
9 – 11th grade	648	10.4	9.3	9.9	(9.5, 10.3)
High school grade/GED or equivalent	1027	20.4	8.8	9.3	(9.0, 9.6)
Some college or AA degree	1461	32.1	9.1	9.7	(9.4, 10.0)
College graduate or above	1239	31.5	9.2	9.7	(9.4, 10.1)
Missing	2	-			
PIR					
< 0.9	1920	17.1	9.6	10.3	(9.9, 10.6
0.9-1.65	1738	19.0	9.4	10.3	(10.1, 10.5
1.66-3.64	1772	27.3	9.4	9.9	(9.6, 10.2
≥ 3.65	1788	36.6	9.1	9.6	(9.4, 9.8)
Missing	502	-			
Race/ethnicity					
Non-Hispanic White	2411	63.6	9.1	9.6	(9.4, 9.8)
Non-Hispanic Black	2162	12.2	8.3	8.9	(8.7, 9.1)
Mexican-American	1058	9.6	10.7	11.3	(11.0, 11.6)
Other Hispanic	820	6.8	10.0	10.5	(9.9, 11.1)
Asian	975	4.8	11.9	12.6	(12.2, 13.0)
Other/multiracial	294	3.0	10.1	10.6	(9.9, 11.3)
BMI^b^					
Underweight	197	2.2	9.3	9.8	(8.9, 10.6)
Normal	3184	37.6	9.4	10.0	(9.8, 10.2)
Overweight	1928	29.7	9.1	9.6	(9.4, 9.8)
Obese	2192	30.5	9.4	10.1	(9.8, 10.3)
Missing	219	-			
Pregnancy status^c^					
Pregnant women	51	4.7	11.9	12.5	(11.3, 13.8)
Non preg./could not ascertain	1034	95.3	10.2	10.8	(10.5, 11.2)
Menopausal status^d^					
Premenopausal	1374	56.9	10.1	10.8	(10.5, 11.1)
Postmenopausal	1087	43.1	9.2	9.8	(9.4, 10.2)

AA: Associate degree; GED: General educational development.

^a^for individuals 20–80 years.

^b^for individuals 2–80 years.

^c^for women 20–44 years.

^d^for women 20–80 years.

**Table 2:** **Percentile distribution, minimum and maximum of blood Mn levels for females, males and both sexes (μg/L); weighted statistics, NHANES 2011-2012**

	Blood Mn concentration (μg/L)		
	Females	Males	All
Min	2.7	1.6	1.6
Percentile 5	5.8	5.3	5.5
Percentile 25	7.9	7.2	7.5
Percentile 50	9.7	8.8	9.2
Percentile 75	12.4	10.6	11.4
Percentile 95	17.8	14.9	16.6
Max	62.5	45.5	62.5

**Figure 4:**
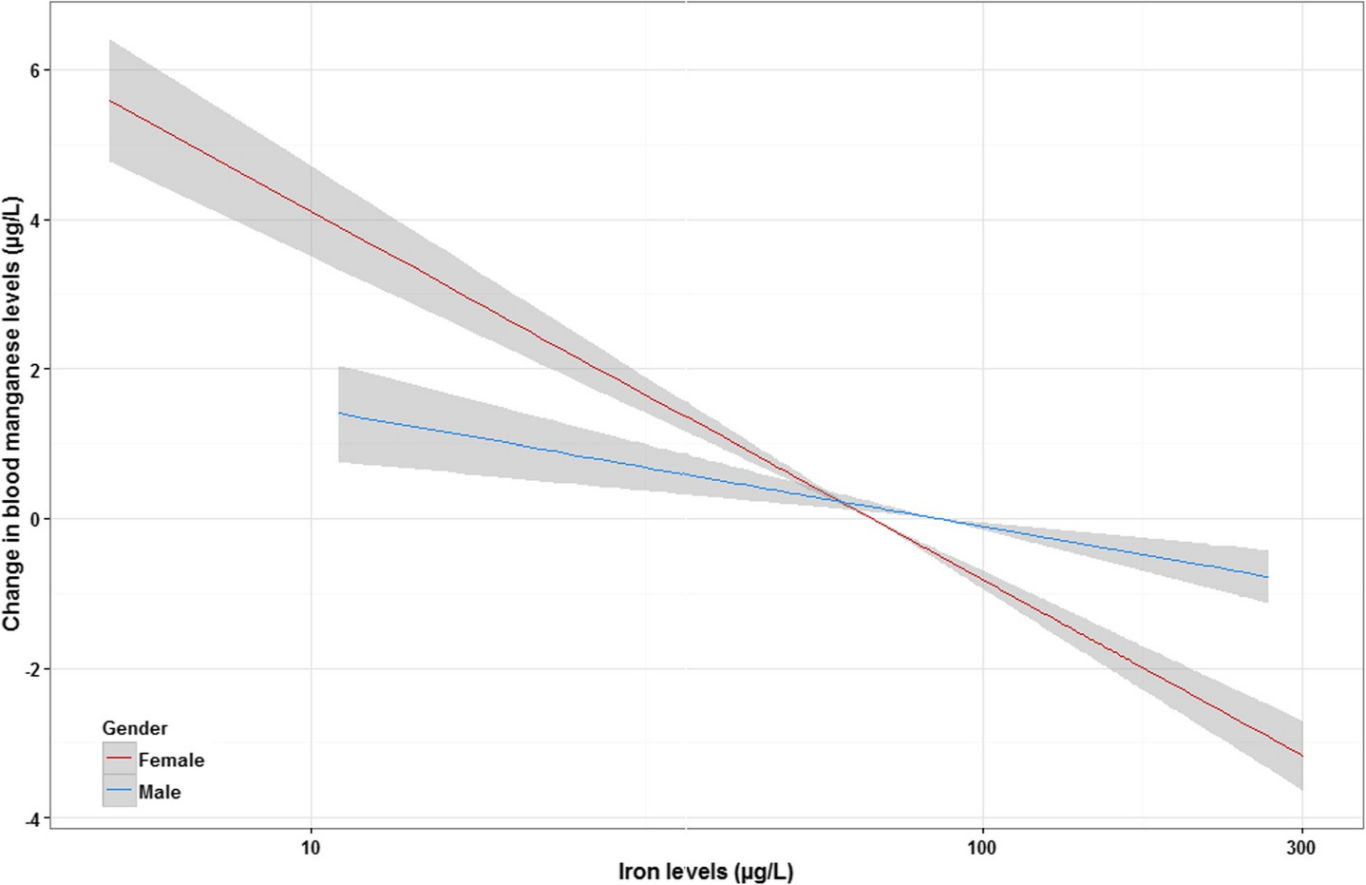
**Sex-stratified association between blood Mn and serum iron levels (95% CI) for individuals 12–80 years, adjusted for age, BMI, PIR, race/ethnicity, and blood lead, cadmium, selenium, and mercury levels (n = 5287).** Statistics are weighted.

In the older age group, i.e. 12 to 80 years, race/ethnicity and blood iron levels were significantly associated with blood Mn levels. In addition, age and sex were marginally associated with Mn levels (both at p = 0.06). Blood Mn levels were lower with older age, higher in women than men, highest in individuals of Asian origin and lowest in non-Hispanic black participants, and higher with lower blood iron level. PIR, BMI, blood lead, mercury, selenium, and cadmium were not associated with blood Mn levels. In a model restricted to individuals aged 20 years and older for whom data on education was available, this variable was not associated with blood Mn levels. In sex-stratified analyses, race/ethnicity, blood iron levels, and age were significantly associated with blood Mn levels in both men and women. Furthermore, we observed that sex acted as an effect modifier in the association between blood iron and Mn levels (p_interaction_ = 0.005). The association estimate was larger among women than men (β for a 10-fold increase in iron levels = −1.57 (95% CI:-2.30, −0.85) and −4.92 (95% CI:-5.64, −4.20), respectively in men and women) (see Figure 4).

**Figure 5:**
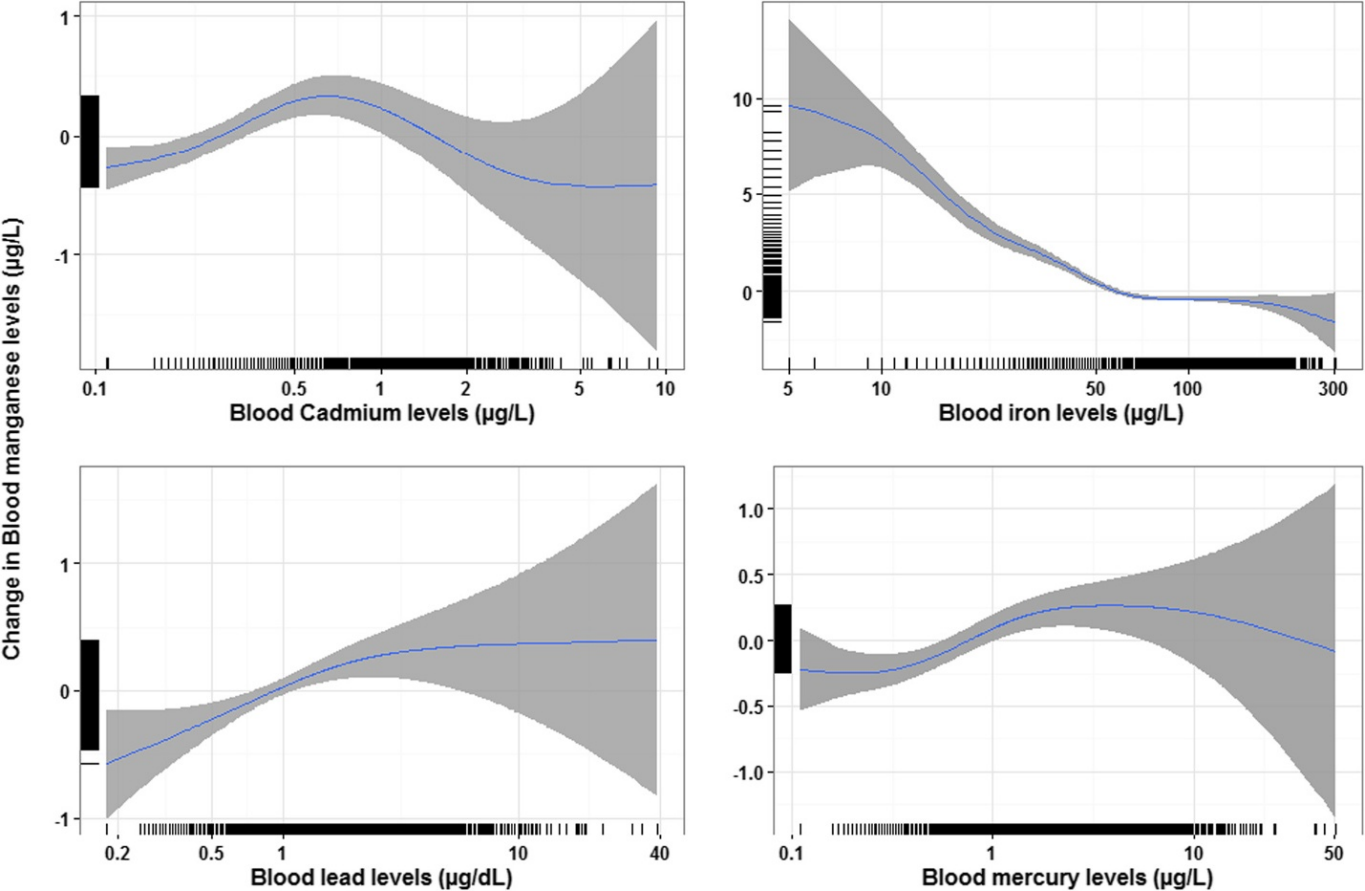
**Association between blood Mn and lead, cadmium, iron, and mercury levels (95% CI) for individuals 12–80 years, adjusted for age, sex, BMI, PIR, race/ethnicity, and blood selenium levels (n = 5287).** Statistics are weighted.

Continuing with the older age group, i.e. 12 to 80 years, we found non-linear relationships between blood Mn levels and cadmium, iron, lead, and mercury levels using GAMs (Figure 5). The relationship between blood Mn and cadmium levels resembled an inverse U-shape with an inflexion point around cadmium concentrations between 0.6 and 0.8 μg/L. Mn levels decreased steadily with higher blood iron levels and reached a plateau around iron concentrations of 75 μg/L. Mn levels increased until mercury levels of 3 μg/L and then remained stable. Finally, blood Mn levels increased with higher blood lead levels, with a sharper slope below lead concentrations of 2.5 μg/dL.

A model restricted to women ages 20 to 44 years (n = 1085) showed that pregnant women had significantly higher blood Mn levels than non-pregnant women (p = 0.04), after adjusting for age, race/ethnicity, blood lead, cadmium, mercury, calcium, selenium, and iron levels. Fifty-one women were pregnant in the study group; their mean adjusted blood Mn level was: 13.1 μg/L compared with 11.2 μg/L for non-pregnant women, the mean adjusted difference was 2.11 μg/L (95% CI: 0.84, 3.38). Finally, premenauposal and postmenauposal women had similar blood Mn levels (adjusted means 10.9 μg/L [95% CI: 10.4 to 11.4], and 10.8 μg/L [95% CI: 10.2 to 11.3], respectively; p for difference = 0.48).

We conducted sensitivity analyses imputing missing data for PIR and BMI, and these yielded results similar to those from our primary analyses. The association estimates for all analyses changed by less than 10% (Results not shown).

## Discussion

The present study is the first report on blood Mn levels in a sample representative of the U.S. population. The characteristics significantly associated with higher blood Mn levels were being female sex, younger age, and Asian origin. In addition, higher blood cadmium levels were associated with higher blood Mn levels in individuals younger than 12 years but not in older individuals. Higher blood Mn levels were also associated with lower blood iron levels, in individuals older than 12 years of age, and we could not examine this relation in younger individuals because the data was not available. Furthermore, pregnant women had higher Mn levels than non-pregnant women. Finally, we observed non-linear relationships between blood Mn and iron, cadmium, mercury, and lead levels.

Females had higher levels than males at all ages, although the difference was smaller during childhood than during adolescence and adulthood (except for 1-years olds, but the number of subjects was small and estimates were imprecise). This suggests that there are sex-related metabolic differences in the homeostatic mechanisms regulating blood Mn levels. A study of dietary uptake of Mn showed that, when consuming a diet adequate in Mn, women might absorb significantly more Mn than men [28]. This result might also be due to the lower iron levels in women that are associated with increased Mn absorption. Furthermore, the high blood Mn levels observed among pregnant women may reflect increased physiological needs for this nutrient during fetal growth. Indeed, maternal blood Mn concentrations increase during pregnancy because of increased fetal demand and Mn accumulation through active transport across the placenta [29].

The homeostatic mechanisms regulating Mn excretion are not fully developed in children, and concerns have been raised that children would thus be more susceptible to overexposure [30]. Thus, the highest concentrations of Mn in infants observed in this study are not surprising since they have increased absorption of Mn through the digestive tract compared to adults [31]. Breast milk contains very low concentrations of Mn, whereas levels vary widely in infant formulas; high levels have been reported in formulas made of soybean and rice [32, 33]. Unfortunately, in the present study the data on consumption of these foods was not available.

To our knowledge, the only other nationally representative population survey analyzing predictors of blood Mn levels was conducted in South Korea, but only adults over 20 years of age were included (KNHANES, n = 2005) [12]. The higher blood Mn levels observed in women than men reported here was also reported in the KNHANES [12]. Furthermore, blood Mn levels in the KNHANES were similar to the levels found in participants of Asian origin of the U.S. NHANES (GM 12.9 and 11.9 μg/L, respectively), which is higher than the average of participants (GM 9.3 μg/L). Dietary differences between resident from the two countries could contribute explaining this finding, such as consumption of tea which has a very high content in Mn [34]. Some findings from the KNHANES differed from the present study. For instance, they reported no age-related differences in blood Mn levels. However, the greatest age-related difference here was for the elevated levels found in children, and the Korean study included only adults. Also, unlike the present study, the Korean study reported significant differences with respect to education, as well as significantly lower levels among postmenauposal women compared with premenauposal women [10]. A factor that could explain discrepancies between the two studies is that findings in the Korean survey were adjusted for serum ferritin levels, which were significantly inversely associated with blood Mn.

In the present study, we were not able to adjust for serum ferritin in our models because it was not measured in this iteration of NHANES. However, we were able to adjust for serum iron levels, and found a significant inverse association with Mn levels only in individuals with iron concentrations below 75 μg/L. This inverse association was also more pronounced among women than for men. Although blood iron concentration is not the best indicator of iron stores and is not a sensitive measure of iron deficiency - unless it is used in combination to total iron-binding capacity measurements - this result might indicate that uptake and transport of Mn are associated with iron status as previously reported in young women [35]. This result is similar to the one previously found in a community based study in Canada [7], and in the Korean study with higher Mn levels in individuals with iron deficiency [12].

Finally, Mn levels were also associated with other trace elements such as lead and mercury. Although we found no evidence in the literature to support our finding, this might reflect common exposure through food, or shared biological mechanisms.

## Conclusion

In this population-based study, we observed higher blood Mn levels in females, younger individuals, participants from Asian background, and in pregnant women. The higher blood Mn levels observed among females suggest sex-related metabolic differences in the regulation of Mn, and elevated levels among pregnant women suggest an important role of Mn in reproduction.

The present study supports the need to take into consideration age- and sex-related differences in blood Mn levels when examining Mn essentiality or toxicity.
